# (−)-Hydroxycitric acid reduced fat deposition via regulating lipid metabolism-related gene expression in broiler chickens

**DOI:** 10.1186/s12944-016-0208-5

**Published:** 2016-02-24

**Authors:** Jing Han, Longlong Li, Dian Wang, Haitian Ma

**Affiliations:** Key Laboratory of Animal Physiology and Biochemistry, College of Veterinary Medicine, Nanjing Agricultural University, Nanjing, 210095 China; School of Life Science and Technology, China Pharmaceutical University, Nanjing, 210009 China

**Keywords:** (−)-Hydroxycitric acid, Lipid metabolism, Broiler chickens

## Abstract

**Background:**

Chicken as a delicious food for a long history, and it is well known that excess fat deposition in broiler chickens will not only induced metabolic diseases, but also lead to adverse effect in the consumer’s health. (−)-Hydroxycitric acid (HCA), a major active ingredient of *Garcinia Cambogia* extracts, had shown to suppress fat accumulation in animals and humans. While, the precise physiological mechanism of HCA has not yet been full clarified, especially its action in broiler chickens. Thus, this study aimed to assess the effect of (−)-HCA on lipid metabolism in broiler chickens.

**Methods:**

A total of 120 1-day-old broiler chickens were randomly allocated to four groups, with each group was repeated three times with 10 birds. Birds received a commercial diet supplemented with (−)-HCA at 0, 1000, 2000 or 3000 mg/kg, respectively, for a period of 4 weeks *ad libitum*.

**Results:**

Body weight (BW) in the 2000 and 3000 mg/kg (−)-HCA groups was significantly decreased (*P* < 0.05) than that in control group. A significantly decreased of serum triglyceride (TG) and density lipoprotein-cholesterol (LDL-C) content were observed in 3000 mg/kg (−)-HCA group (*P* < 0.05). Broiler chickens supplmented with 2000 and 3000 mg/kg (−)-HCA had pronouncedly higher hepatic lipase (HL) activity, hepatic glycogen and non-esterified fatty acid (NEFA) contents in liver (*P* < 0.05). Serum free triiodothyronine (FT3) and thyroxin (T4) contents were significantly higher in 3000 mg/kg (−)-HCA group (*P* < 0.05) compared with the control group. Supplemental (−)-HCA markedly decreased fatty acid synthase (FAS) and sterol regulatory element binding protein-1c (SREBP-1c) (*P* < 0.05) mRNA levels, while the mRNA abundance of adenosine 5′-monophosphate-activated protein kinaseβ2 (AMPKβ2) (*P* < 0.05) was significantly increased. In addition, ATP-citrate lyase (ACLY) mRNA level (*P* < 0.05) was significantly decreased in broiler chickens supplemented with 3000 mg/kg (−)-HCA. No differences was observed on carnitine palmitoyl transferase-I(CPT-I), while peroxisome proliferators-activated receptor α (PPARα) mRNA level (*P* < 0.05) was significantly increased in broiler chickens supplemented with 2000 and 3000 mg/kg (−)-HCA.

**Conclusions:**

Supplemental (−)-HCA inhibited lipogenesis by inhibiting ACLY, SREBP-1c and FAS expression, and accelerated lipolysis through enhancing HL activity and PPARα expression, which eventually led to the reduced abdominal fat deposition in broiler chickens.

**Graphical abstract:**
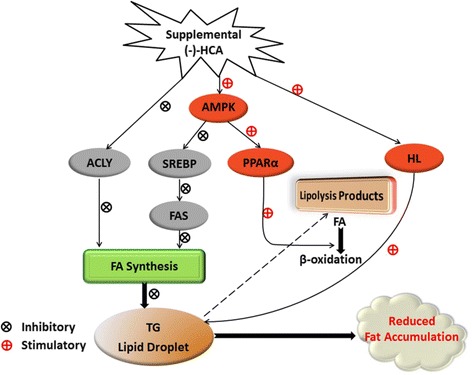
Mechanism of (−)-HCA effect on hepatic lipids metabolism.

## Background

In few decades, the principle aim of poultry production in many coutries has been to increase growth rate. Although intensive genetic selection in broilers has contributed to improve growth rates, modern bird strains often exhibit excessive body fat deposition which is not wanted by most of consumers [1], since coronary heart disease and arteriosclerosis are srongly related to the dietary intake of cholesterol. The production of brolier chickens with excess body fat also has proven to be one of the main problems encoutered within the broiler industry today, as evidenced by significant increased feed costs, decreased final product quality, and a significant economic loss to poultry processing plants [2]. The economic concern and the recognition of consumer aversion to excess fatty tissue deposition have led many poultry scientists to study the factors that are associated with fat deposition and the methods needed to reduce it [2].

(−)-Hydroxycitric acid [(−)-HCA] is the principal acid of fruit rinds of *Garcinia cambogia* [3]. (−)-HCA being a potent inhibitor of ATP-citrate lyase which catalyzes the extramitochondrial cleavage of citrate to oxaloacetate and acetyl-CoA, limits the availability of acetyl-CoA units required for fatty acid synthesis [4, 5]. Many studies demonstrate both *in vitro* and *in vivo* that (−)-HCA suppresses the *de novo* fatty acid synthesis [4, 6]. Animal studies have suggested that (−)-HCA causes weight loss [7, 8]. Moreover, it increases rates of hepatic glycogen synthesis, and decreases body weight gain [9–11]. Even though some studies have been conducted on the regulation of (−)-HCA on lipid metabolism in rats and mice, the underlying mechanisms of this physiological role of (−)-HCA are not fully understood. Importantly, no detailed information is available on the effect of (−)-HCA on lipid metabolism in broiler chickens.

Unliken mammalian species, the liver is the main site of *de novo* fatty acid synthesis in birds, accounting for 95 % of all lipid production in young chicks, with the adipose tissue is served only as a lipid storage site [12, 13]. In consequence, most of the endogenous body lipids are of hepatic origin and the development of adipose tissue depends on the availability of plasma triglycerides that are hydrolyzed prior to their utilization by adipocytes. Numerous studies have indicated that, among the different lipid metabolic pathways that can be altered with fat deposition, liver fatty acid metabolism is the most important [14, 15]. These results prompted researches on gene expression in the liver, especially those gene involved in fatty acid synthesis and secretion [16, 17], including peroxisome proliferators-activated receptor a (PPARα), AMP-activated protein kinase (AMPK), fatty acid synthase (FAS), sterol regulator element binding protein-1c (SREBP-1c), acetyl CoA carboxylase (ACC), carnitine palmitoyl transferase-I (CPT-I), and ATP-citrate lyase (ACLY). Despite the wealth of information available indicating that (−)-HCA alters lipid metabolism in mice, rats and humuans, little is known about the effect of (−)-HCA on lipid metabolism and hepatic gene expression in poultry, and especially in broiler chickens.

Chicken as a delicious food for a long history, and it is well known that too much abdominal fat deposition in broiler chickens will not only induced broiler ascites syndrome, sudden death and other metabolic diseases, but also lead to adverse effect in the consumer’s health. Despite the wealth of information available indicating that (−)-HCA alters lipid metabolism in mice, rats and humans, little is known about the effect of (−)-HCA on lipid metabolism and hepatic gene expression in poultry, and especially in broiler chickens. Therefore, the aim of present study was to explore the effect of (−)-HCA on lipid metabolism and hepatic lipid metabolism-related genes expression in broiler chickens which might help to identify the possible mechanism of (−)-HCA in decreasing fat deposition in adipose tissue.

## Results

### Growth performance

As shown in Table 1, no statistically significant differences among average daily feed intake (ADFI) and feed conversation effeciency were observed in brolier chickens, whereas body weight (BW) and average daily gain (ADG) in the 2000 mg/kg and 3000 mg/kg (−)-HCA groups were significantly lower than in the control group (*P* < 0.05).

**Table 1:** Effect of (−)-HCA on growth performance of broiler chickens from 22 to 49d of age^a^

Item^b^	Supplemental (−)-HCA (mg/kg)			
	0	1000	2000	3000
BW (g per birds) 49 d	3460.23 ± 49.22	3360.03 ± 52.04	3337.23 ± 42.53*	3312.83 ± 28.09*
ADG (g per birds per d) 22 to 49 d	86.51 ± 1.36	83.51 ± 1.44	82.83 ± 1.16*	82.10 ± 0.73*
ADFI (g per birds per d) 22 to 49 d	177.95 ± 6.92	174.48 ± 7.28	164.22 ± 6.58	171.57 ± 7.19
Feed conversion ratio (g :g) 22 to 49 d	2.06 ± 0.08	2.09 ± 0.09	1.98 ± 0.08	2.09 ± 0.09

^a^ BW = body weight; ADG = average daily gain; ADFI = average daily feed intake

^b^ Data in each group represent mean values of three replicates, one replicate indicating the average of 25 chickens in one cage. Values are means ± SE. * *P* < 0.05, compared with the control group

### Carcass composition

The effect of supplemental (−)-HCA on the relative weight of abdominal fat, liver, leg muscle and pectoral muscle in brolier chickens were shown in Table 2. Althought there were no statistically significant differences (*P* > 0.05), the aboslute and relative abdominal fat weight among all experimental groups tended to be lower than in the control group. No significant difference was observed in the relative weight of leg muscle and pectoral muscle (*P* > 0.05), a marked decreased in the aboslute and relative liver weight were noted in the 3000 mg/kg (−)-HCA group (*P* < 0.05).

**Table 2:** Effect of (−)-HCA on carcass composition in broiler chickens

Item	Supplemental (−)-HCA (mg/kg)			
	0	1000	2000	3000
Absolute abdominal fat (g)	103.39 ± 4.62	95.66 ± 6.60	94.55 ± 5.12	92.33 ± 4.41
Relative abdominal fat (%)	2.97 ± 0.12	2.80 ± 0.20	2.90 ± 0.15	2.90 ± 0.14
Absolute liver weight (g)	57.57 ± 3.03	55.52 ± 1.55	50.01 ± 1.2*	48.94 ± 2.21*
Relative liver weight (%)	1.65 ± 0.07	1.65 ± 0.04	1.57 ± 0.04	1.47 ± 0.04*
Relative pectoral weigh (%)	13.88 ± 0.19	14.09 ± 0.24	13.93 ± 0.11	13.49 ± 0.18
Relative leg weight (%)	8.50 ± 0.19	8.39 ± 0.07	8.57 ± 0.10	8.63 ± 0.12

Values are means ± SE. Each (−)-HCA treated group represent 12 chickens at the age of 49 days, * *P* < 0.05, compared with the control group

### Lipid metabolic parameters

The serum and hepatic TG contents were decreased, and a significant decrease was observed in the 3000 mg/kg (−)-HCA treatment group (*P* < 0.05) (Tables 3 and 4). Although no noticeable change was observed in the serum NEFA concentration (Table 3), while the NEFA concentration in liver was significantly increased (*P* < 0.05) in the 2000 mg/kg and 3000 mg/kg (−)-HCA groups than in the control group (Table 4). Serum LDL-C content was significantly lower in broiler chickens fed with 3000 mg/kg (−)-HCA than in the control group (*P* < 0.01), while no noticeable change was observed in the serum TC and HDL-C content following treatment with (−)-HCA compared to the control group (Table 3). Broiler chickens fed with 2000 mg/kg and 3000 mg/kg (−)-HCA resulted in a pronounced higher level of HL activity as compared to the control group (*P* < 0.01) (Table 4). Compared with the control group, the hepatic glycogen content also was significantly increased (*P* < 0.01) in broiler chickens fed 2000 mg/kg and 3000 mg/kg (−)-HCA (Table 4).

**Table 3:** Effect of (−)-HCA on serum lipid parameters in broiler chickens

Item	Supplemental (−)-HCA (mg/kg)			
	0	1000	2000	3000
TC (mmol/L)	3.66 ± 0.08	3.83 ± 0.22	3.42 ± 0.22	3.22 ± 0.14
TG (mmol/L)	0.56 ± 0.04	0.51 ± 0.04	0.48 ± 0.03	0.19 ± 0.01**
LDL-C (mmol/L)	0.87 ± 0.13	0.8 ± 0.1	0.72 ± 0.15	0.46 ± 0.06*
HDL-C (mmol/L)	1.72 ± 0.09	1.69 ± 0.07	1.68 ± 0.07	1.87 ± 0.10
NEFA (μmol/L)	1401.43 ± 110.94	1267.83 ± 77.74	1390.03 ± 141.97	1437.93 ± 56.08

Values are means ± SE. Each (−)-HCA treated group represent 12 chickens at the age of 49 days. * *P* < 0.05, ** *P* < 0.01 compared with the control group

**Table 4:** Effect of (−)-HCA on liver homogenates concentration of triglycerides (TG), hepatic lipase (HL), non-esterified fatty acid (NEFA)

Item	Supplemental (−)-HCA (mg/kg)			
	0	1000	2000	3000
TG (mmol/g liver)	3.31 ± 0.56	2.78 ± 0.40	2.75 ± 0.58	2.02 ± 0.34*
NEFA (μmol/L)	65.83 ± 5.66	95.41 ± 9.52	109.90 ± 19.21*	125.52 ± 12.37**
HL (U/mL)	1.57 ± 0.11	1.59 ± 0.09	3.41 ± 0.27**	2.68 ± 0.28**
Hepatic glycogen (mg/g liver)	2.63 ± 0.23	3.63 ± 0.34	3.91 ± 0.41*	3.91 ± 0.65*

Values are means ± SE. Each (−)-HCA treated group represent 12 chickens at the age of 49 days. * *P* < 0.05, ** *P* < 0.01 compared with the control group

### Serum metabolic hormones

As shown in Table 5, serum FT_3_ and T_4_ contents were significantly higher in the 3000 mg/kg (−)-HCA group than in the control group (*P* < 0.05). However, serum insulin and glucagon concentration were not affected by the addition of (−)-HCA (*P* > 0.05).

**Table 5:** Effect of (−)-HCA on serum metabolic hormones in broiler chickens

Item	Supplemental (−)-HCA (mg/kg)			
	0	1000	2000	3000
Insulin (pg/mL)	13.17 ± 0.87	12.85 ± 0.47	11.56 ± 1.03	13.05 ± 0.74
Glucagon (pg/mL)	496.56 ± 24.57	422.86 ± 21.10	484.43 ± 25.02	464.29 ± 47.38
T_3_ (ng/mL)	1.19 ± 0.05	1.21 ± 0.05	1.13 ± 0.06	1.37 ± 0.14
T_4_ (ng/mL)	42.82 ± 0.92	42.25 ± 1.58	42.54 ± 1.25	47.65 ± 1.53*
FT_3_ (fmol/ml)	2.04 ± 0.15	1.98 ± 0.12	2.16 ± 0.21	3.33 ± 0.65*
FT_4_ (fmol/ml)	6.41 ± 0.21	6.86 ± 0.27	6.31 ± 0.25	6.70 ± 0.26

Values are means ± SE. Each (−)-HCA treated group represent 12 chickens at the age of 49 days. * *P* < 0.05 compared with the control group

### Hepatic lipid metabolic gene expression

**Fig. 1:**
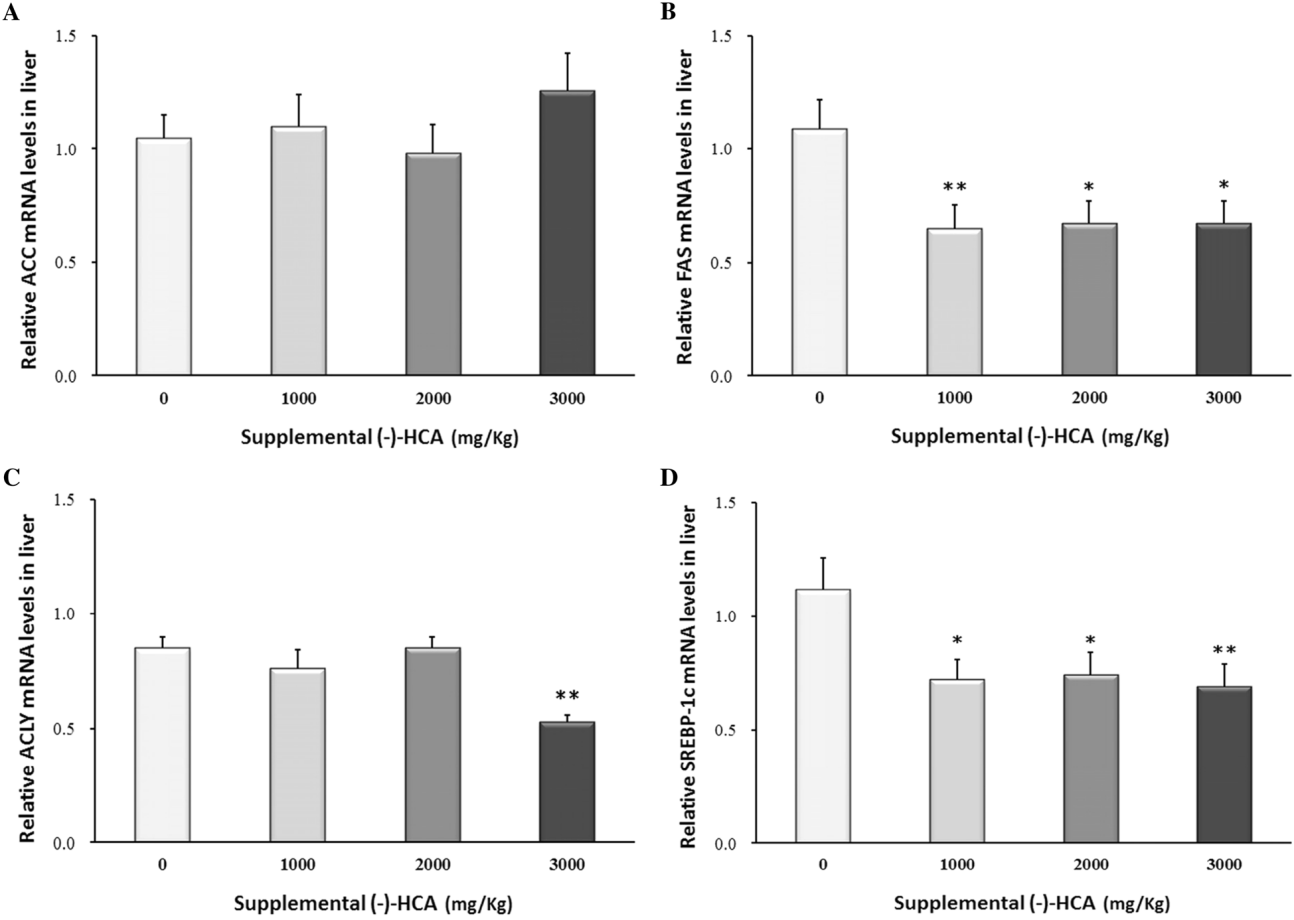
Effect of (−)-HCA on (**a**) ACC, (**b**) FAS, (**c**) ACLY and (**d**) SREBP-1c mRNA levels in the liver of broiler chickens. Values are means ± SE. (12 chickens per treatment are representative of four birds per replicate). * *P* < 0.05, ** *P* < 0.01 compared with the value of 0 mg of (−)-HCA/kg group

**Fig. 2:**
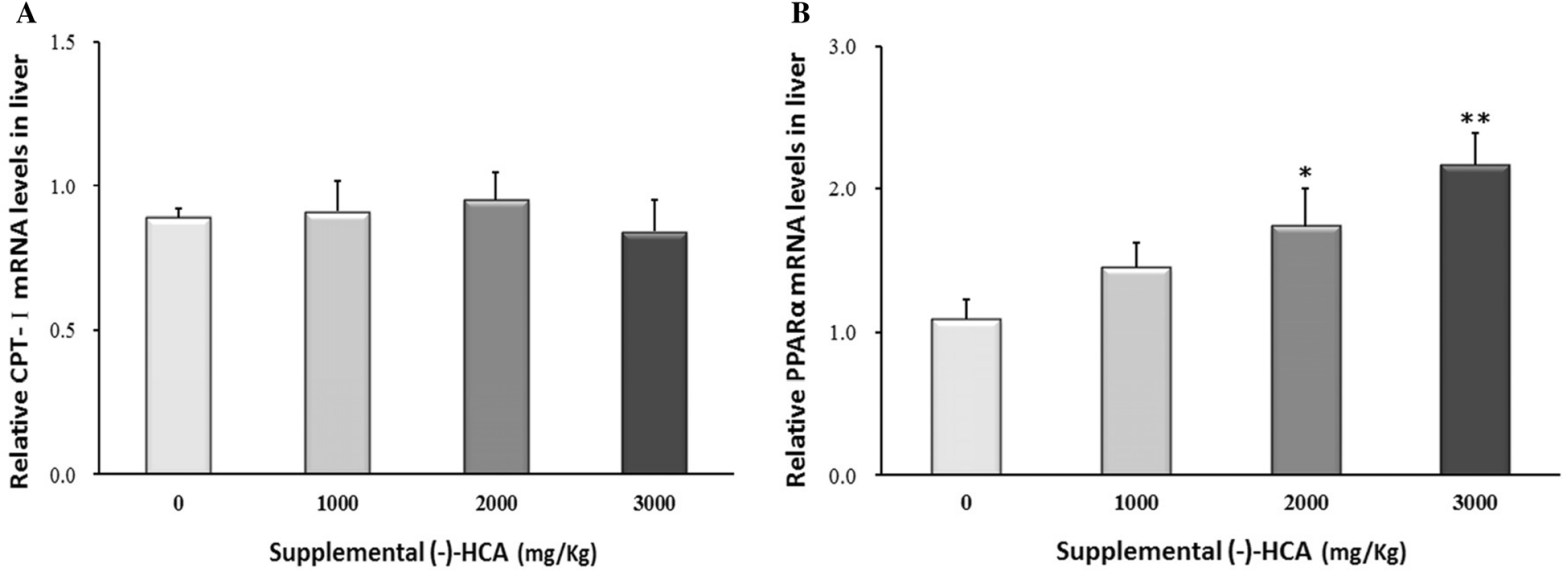
Effect of (−)-HCA on (**a**) CPT-I and (**b**) PPARα mRNA levels in the liver of broiler chickens. Values are means ± SE. (12 chickens per treatment are representative of four birds per replicate). * *P* < 0.05, ** *P* < 0.01 compared with the value of 0 mg of (−)-HCA/kg group

**Fig. 3:**
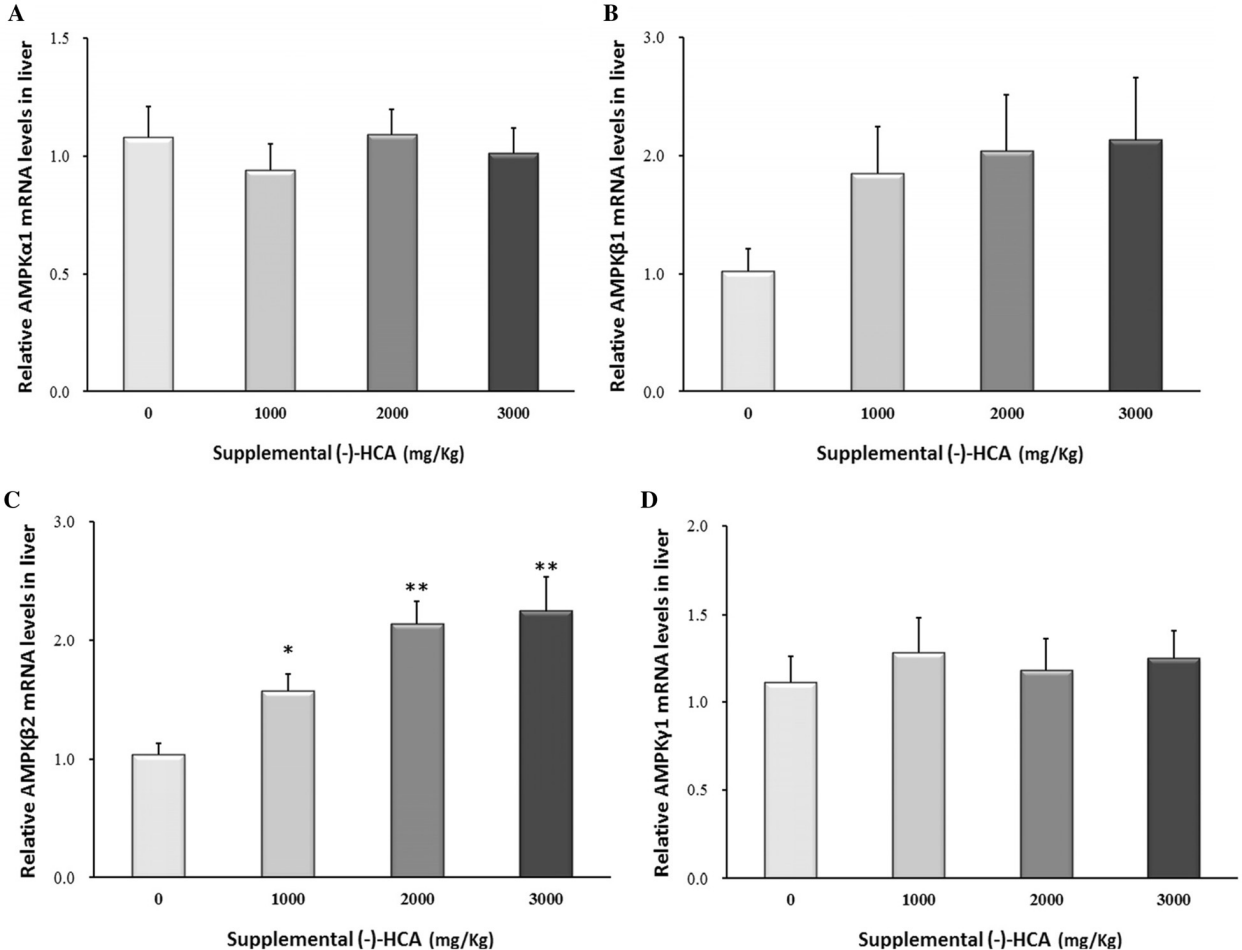
Effect of (−)-HCA on (**a**) AMPKα1, (**b**) AMPKβ1, (**c**) AMPKβ2 and (**d**) AMPKγ1 mRNA levels in the liver of broiler chickens. Values are means ± SE. (12 chickens per treatment are representative of four birds per replicate). * *P* < 0.05, ** *P* < 0.01 compared with the value of 0 mg of (−)-HCA/kg group

Supplemental of (−)-HCA markedly decreased the FAS and SREBP-1c mRNA levels (*P* < 0.05) (Fig. 1b and d). The mRNA level of ACLY (*P* < 0.05) was significantly decreased in broiler chickens supplemented with 3000 mg/kg (−)-HCA (Fig. 1c), whereas the mRNA abundance of ACC (Fig. 1a) was unaffected by (−)-HCA (*P* > 0.05). Although no significant differences were observed in CPT-I (*P* > 0.05) gene expression (Fig. 2a), while the mRNA level of PPARα (*P* < 0.05) was significantly increased in broiler chickens supplemented with 2000 mg/kg and 3000 mg/kg (−)-HCA groups than that in the control group (Fig. 2b). Futhermore, no noticeable change was observed in the levels of AMPKα1, AMPKβ1 and AMPKγ1 gene expression (Fig. 3a, b and d), but the mRNA abundance of AMPKβ2 (*P* < 0.05) was significantly higher in broiler chickens supplemented with (−)-HCA groups than that in the control group (Fig. 3c).

## Discussion

Dyslipidemia is a main cardiovascular risk factor for coronary heart disease incidence and mortality [18]. Lipid metabolism disorders can accelerate the atherosclerosis process and its consequences, such as heart failure and coronary atherosclerosis [18]. Even though some drugs could be used to therapy these diseases, there would be some adverse effects at the time. It well known that “nutraceutical” products, i.e., a food (or part of a food) that provides medical or health benefits, including the prevention and/or treatment of a disease [18]. Nutraceuticals are effectively able to reduce the burden of the atherosclerosis process and coronary heart disease [19, 20]. Also, many studies have evaluated the potential role of nutraceuticals in the prevention of dyslipidaemia both in animal models [21, 22] and in humans [23, 24]. (−)-Hydroxycitric acid (HCA), an extract from the dried fruit rind of *Garcinia cambogia*, is widely used as an ingredient for nutritional supplements to reduce food intake, appetite and body weight. In these studies, (−)-HCA administration led to the loss of body weight and appetite reduction [25, 26], decreased energy intake [27] and increased fat oxidation [28]. Chicken as a delicious food for a long history, and it is well known that too much abdominal fat and subcutaneous fat deposition in broiler chickens will not only induced broiler ascites syndrome, sudden death and other metabolic diseases, but also lead to adverse effect in the consumer’s health. Despite the wealth of information available indicating that (−)-HCA alters lipid metabolism in mice, rats and humans, little is known about the effect of (−)-HCA on lipid metabolism and hepatic gene expression in poultry, and especially in broiler chickens.

In the present study, broiler chickens fed with (−)-HCA-containing diet for 28 days had reduced body weight and average daily gain as compared to a non-(−)-HCA control group. In addition, the absolute and relative abdominal fat weight among all experimental groups tended to be lower than in the control group, and a marked decreased in the absolute and relative liver weight were noted in the 3000 mg/kg (−)-HCA group. As a weight loss agent, many studies had report that (−)-HCA safely promotes weight loss in laboratory animals [8, 29] and humans [26, 30], and suggest food intake regulation may be a major mechanism for weight loss induced by (−)-HCA [8, 31]. However, no statistically significant differences among average daily feed intake were observed in brolier chickens supplemental with (−)-HCA. The results obtained here was similar to Sebola et al., who reports that *Garcinia cambogia* leaf supplementation has no effect on feed intake in the finisher stage of male Ross 308 broiler chickens [32]. This discrepancy may be due to the different kind of animal used in those studies. Present results indicated that the suppressive effect of (−)-HCA on body weight in broiler chickens is not *via* regulating the feed intake. Also, it is well known that the decrease in body weight gain is caused not only by a decrease in food intake but also an increase in energy expenditure (EE). Previous study shows that (−)-HCA could enhance energy expenditure in rats [33]. Although we did not measure energy expenditure, but no change in feed intake provides indirect evidence for that broiler chickens of these groups supplemental with (−)-HCA were not less efficient in using the food energy for the accretion of body mass, which suggests that energy expenditure was increased. Thyroid hormones, including T_3_ and T_4_, are recognized as key metabolic hormones, with T_3_ being the most functionally active form. The serum level of thyroid hormones is associated with energy production [34, 35]. In the present study, FT_3_ and T_4_ were elevated in the 3000 mg/kg (−)-HCA supplemented group. As observed previously [36], reduce of circulating thyroid hormone levels lowers the metabolic rate as a protective mechanism for the body’s energy reserves. Chen et al. also reported that broiler chickens receiving experimental diet with creatine pyruvate had greater energy expenditure, accompanying by an elevated the level of serum FT_3_ [37]. The suppressive effect of (−)-HCA on body weight regain is not only due to a reduction in food intake, presumably also increased thermogenesis [8]. Thus, we believed that different from mammals, the suppressive effect of (−)-HCA on body weight might be regulated not only by the feed intake, but also through increasing thyroid hormones level, which enhanced energy expenditure in broiler chickens.

Previous studies had presume that an increased fatty acid oxidation and decreased fat accumulation in liver of animal with (−)-HCA-treated contributed to decrease its weight [5, 38]. Thus, we presume that another mechanism of (−)-HCA reduced body weight could due to it inhibition the fat deposition in broiler chicken. Present study showed that the relative liver weight was significantly decreased in birds treated with (−)-HCA. This result was similar to the findings of Saito et al. who observed that *Garnicia cambogia* leaf supplementation reduced weight gain and liver weight of rats [39]. Kim et al. also reports that the relative liver weight was reduced in rats fed with a mixture composed of *Garcinia cambogia* extract, soypeptide, and L-carnitine [40]. Although previous studies [4, 5, 38] presume that fatty acid oxidation increase and fat accumulation decrease in liver of (−)-HCA-treated animal contributed to weight loss, while the physiological explanation is not clear and merits further investigation. It is reported that (−)-HCA inhibits ATP-citrate lyase and increases the cellular pool of citrate, which in turn inhibits glycolysis and thus redirects the carbon atoms for glycogen production [41]. Although serum insulin and glucagon concentration were not affected by the addition of (−)-HCA, our data showed that the hepatic glycogen content was significantly higher in the (−)-HCA groups than that of control group. *In vitro* and *in vivo* studies showed that (−)-HCA inhibits the *de novo* fatty acid synthesis and increases rates of glycogen synthesis [42–45]. (−)-HCA has a structure similar to citrate, which is generally known as an allosteric regulator for a number enzymes that are involved in carbohydrate and fat metabolism [46]. Therefore, administration of (−)-HCA is expected to cause metabolic ramification. Once glucose enters intracellular space across sarcolemma, it is rapidly phosphorylated to glucose 6-phosphate and converted into glycogen through the glycogen synthesis pathway and lactate through the glycolytic pathway. (−)-HCA has been found to inhibit phosphofructokinase, a key enzyme controlling glycolysis [47]. Thus, inhibition of the glycolysis pathway by (−)-HCA divert more glucose 6-phosphate into the glycogen synthesis pathway in liver. Taking account of those results, an alternative mechanism could be proposed that the (−)-HCA effect on increased rate of glycogen synthesis probably was due to its inhibitory effect on the glycolytic pathway.

Althought there were no statistically differences, the absolute and relative abdominal fat weight were tended to be lower in (−)-HCA fed broiler chickens. Similar resutls was repoted by Sebola et al., that daily supplemention with 300 mg of *Garcinia cambogia* leaf reduced fat pad weight by 18.75 % in male Ross 308 broiler chickens [32]. High doses of *Garcinia cambogia* extract are effective in suppressing fat accumulation in developing male Zucker obese rats [39]. It is well known that (−)-HCA supplementation reduces the amount of carbohydrates that are converted to body fat [44] and blocks the enzyme ATP-citrate lyase that acts as a key building block for fat synthesis. Thus, more carbohydrates will be stored as glycogen within the liver or muscle. Such transient changes in glycogen storage level do not appear to alter feed intake in broiler chickens. Therefore, it appears here that broiler chickens would likely benefit practically from (−)-HCA in terms of suppression of body fat accumulation and as such help to increase demand by consumers. Present study also found that serum TG and LDL-C content were depressed by addition of 3000 mg/kg (−)-HCA. The result was in agreement with the result of Preuss et al., who reports that after eight weeks groups treated with (−)-HCA showed significant decrease in low density lipoprotein and triglycerides levels than in the placebo group [25]. Haymamizu also reports that serum TG is lower in mice treated with 3.3 % *Garcinia cambogia* extract [48]. Recently, a double-blind randomized 12 week trial showed significant reductions in total cholesterol and low-density lipoprotein cholesterol in the *Garcinia cambogia* group, compared to the placebo group [49]. The liver is the primary site of fatty acid synthesis in poultry [50]. In the present study, supplementation of 2000 or 3000 mg/kg (−)-HCA in broiler chickens improved the liver NEFA level. In contract, the liver TG level declined in the treatment group. These results were expected, since (−)-HCA is known to inhibit ATP-citrate lyase which decreases the transformation of citrate into acetyl CoA, an essential building block for cholesterol and triglyceride biosynthesis [51]. Less acetyl CoA available for triglyceride synthesis in broiler chickens treated with (−)-HCA, may result in a decreased concentration of liver TG. It is reasonable to speculate that (−)-HCA directly stimulated the β-oxidation pathway in hepatocytes, catalyzed the conversion of TG to glycerol and fatty acid, and increased the hepatic uptake of the released NEFA [52, 53]. In addition to the above liver lipid metabolic parameters, HL is another important lipid metabolic enzyme produced primarily by the liver [54]. This study demonstrated a pronounced increase in HL activity after administration of 2000 or 3000 mg/kg (−)-HCA in broiler chickens, which result in decreased lipid synthesis [54, 55]. From the above finding, we speculated that (−)-HCA supplemental in diet not only inhibited triglyceride biosynthesis but also accelerated lipolysis, which in turn decreased the abdominal fat deposition in broiler chickens.

Many studies had certified that (−)-HCA could inhibit ATP-citrate lyase, an enzyme which plays a crucial role in energy metabolism during *de novo* lipogenesis [5, 26, 29, 30, 39]. In our present study, the expression of ATP-citrate lyase (ACLY) mRNA was lower in treatment group than that in control group, which indicated that (−)-HCA, similar to it action on rats and human, also regulated *de novo* lipogenesis in broiler chickens by inhibiting ATP-citrate lyase. Fat accumulation is complex processes that are characterized by many changes in expression of genes controlling lipogenesis and lipolysis. ATP-citrate lyase catalyzes the extramitochondrial cleavage of citrate to oxaloacetate and acetyl-CoA. Acetyl-CoA, which were catalyzed by acetyl CoA carboxylase (ACC) and fatty acid synthase(FAS), is used for the fatty acid synthesis. Although there was no observed difference in the level of ACC mRNA expression in our study, supplement of (−)-HCA markedly decreased levels of FAS. Also, we found that the level of SREBP-1c gene expression was lower than that in control group after administration of (−)-HCA. SREBP (sterol response element binding protein), the transcription factor of the leucine zipper family, have been described as regulators of biosynthesis of cholesterol and fatty acids in the liver [56]. SREBP-1c is preferentially involved in the activation of genes that control the synthesis of fatty acid [57]. As one potential regulators, SREBP-1c can directly stimulate the transcription of genes encoding FAS enzymes [58, 59]. Another interesting observation in this study was that the expression of AMPKβ2 was increased in the (−)-HCA treated groups. AMP-activated protein kinase (AMPK) has been implicated as a key regulator of physiological energy dynamics, including glucose transport and lipolysis [60], AMPKβ2 is a regulatory subunit of the AMPK [61]. The inhibition of FAS expression by AMPK activation was previously reported in primary cultured hepatocytes [62, 63]. Also, previous studies shown an inverse correlation between AMPK and SREBP-1c activities in hepatocytes and in livers of re-fed mice and ethanol-fed mice [64, 65]. Recently study shows that Ser^372^ phosphorylation of SREBP-1c by AMPK may contribute to the ability of polyphenols and metformin to inhibit proteolytic cleavage and nuclear translocation of SREBP-1c in hepatocytes under high glucose plus insulin conditions, thereby preventing transcription of target lipogenic genes [66]. Therefore, the significance of the expression changes of AMPKβ2, SREBP-1c, ACLY, and FAC genes indicates that these genes might be directly responsible for the effect of (−)-HCA on fatty acid synthesis and lipogenesis in broiler chickens. However, elucidation of the precise mechanism involved in the modification of these genes by (−)-HCA needs further investigation.

It is hypothesized that (−)-HCA could increase fat oxidation *via* inhibiting malonyl CoA formation, which in turn, would activate carnitine palmitoyl transferase-I (CPT- I) activity [67, 68]. However, no significant differences were observed in the level of CPT-Igene expression in broiler chickens supplemented with (−)-HCA. Treatment of human adipocytes with HCA-SX resulted in a significant down-regulation of 348 and induction of 366 fat- and obesity-related genes such as hormone sensitive lipase, peroxisome proliferator-activated receptors gamma (PPARγ), coactivator 1α, Leptin, and hypoxia-inducible factor-1 genes [69]. As shown in Fig. 2b, (−)-HCA treatment caused a significant up-regulation of peroxisome proliferators activated receptor α (PPARα) expression in broilers liver tissue, which was similar to a study in 3T3-L1 cells, where *Hibiscus* extract (HCA content in *Hibiscus* extract is high) significantly attenuated the expression of PPARα was observed [70]. PPARa is a member of the nuclear hormone receptor family of transcription factors. It is expressed in the liver, heart, skeletal muscle and brown adipose tissue where it regulates the expression of genes that control mitochondrial and peroxisomal fatty acid oxidation [71]. Rocchi and Auwerx has demonstrated that PPARα can increase the rate of fatty acid β-oxidation by increasing the expression of several PPARα target genes [72]. Considering the decreased TG content in serum and liver after (−)-HCA treatment, it is possible that (−)-HCA improved the expression of PPARα in liver and induced transcriptional up-regulation of enzymes involved in the β-oxidation of fatty acids, thus decreasing the TG content.

## Conclusions

**Fig. 4:**
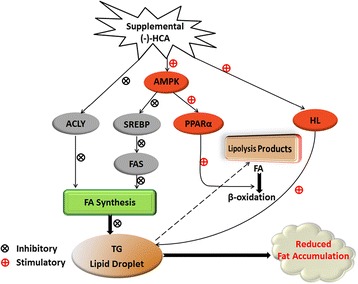
Mechanism of (−)-HCA effect on hepatic lipids metabolism. Supplemental (−)-HCA inhibited lipogenesis by inhibiting ACLY, SREBP-1c and FAS expression, and accelerated lipolysis through enhancing HL activity and PPARα expression, which eventually led to reduced abdominal fat deposition in broiler chickens. The variance of gene expression which involved in lipid metabolism might associate with the mechanism of how (−)-HCA reduced the accumulation of fat in broiler chickens

Present results showed that supplemental with (−)-HCA significantly decreased the body weight, TG content and increased hepatic glycogen content. The SREBP-1c, ACLY and FAS mRNA levels were significantly decreased, while AMPKβ2 and PPARα mRNA levels were significantly elevated in broiler chickens supplemental with (−)-HCA. These results indicated that supplemental (−)-HCA inhibited lipogenesis by inhibiting ACLY, SREBP-1c and FAS expression, and accelerated lipolysis through enhancing HL activity and PPARα expression, which eventually led to reduced abdominal fat deposition in broiler chickens (Fig. 4). The variance of gene expression which involved in lipid metabolism might associate with the mechanism of how (−)-HCA reduced the accumulation of fat in animal.

## Methods

### Extract of Garcinia cambogia

The *Garcinia cambogia* extract containing (−)-HCA was obtained from An Yun Co.Ltd (Zhengzhou, China). The *carcinia cambogia* contain 56-58 % (−)-hydroxycitric acid including its free and lactone form, and it also contains 12-14 % cellulose, 5.5-6 % α-D-melibiose, 2.5-3 % β-D-lactin, 1.5-2 % D-mannopyranose, 11-12 % oxophenic acid, 2-3 % octadecyl alcohol, 3.5-4 % Coenzyme A and 1.5-2 % inorganic elements. The experimental diets contained *Garcinia combogia* at 0, 1.7, 3.4 and 5.1 g/kg diet. These were equivalent of 0, 1000, 2000, 3000 mg/kg (−)-HCA diet.

### Animal and treatment

A total of 120 1-day-old male broiler chickens (Ross 308), obtained from Jiangsu Wuxi chicken breeding company (Wuxi, China), were weighted and allocated to four treatment groups, each of which included three replicates of 10 birds. Broiler chickens were fed on the same basal diets from 1 to 49 d, including starter (1 to 21 d) and finisher (22 to 49 d) rations that were formulated according to NRC (1994) recommendations (Table 6) [73]. At the finisher phase, four groups were supplemented with 0 mg/kg, 1000 mg/kg, 2000 mg/kg or 3000 mg/kg diets (−)-HCA. The animal care and use protocol was approved by the Institutional Animal Care and Use Committee of Nanjing Agricultural University, China.

**Table 6:** Composition and nutrient levels of the experimental diets

Item	Starter	Finisher
Ingredient (%)		
Corn	52.6	57..4
Wheat bran	2.0	4.0
Soybean meal	31.1	27.0
Fish meal	6.0	3.0
Calcium phosphate	1.0	1.5
Limestone	1.2	1.2
DL-Methionine	0.3	0.1
Vitamin-mineral premix^a^	0.5	0.5
Rapeseed oil	5.0	5.0
NaCl	0.3	0.3
Nutrient composition Calculated		
ME^b^ (kcal/kg)	3.10	3.14
CP (%)	22.52	19.74
Lys (%)	1.19	1.08
Met + cystine (%)	0.93	0.71
Ca (%)	1.00	0.9
Total P (%)	0.80	0.76
Available P (%)	0.47	0.39

Nutrient level of the diets was based on National Research Council recommendations

^a^Vitamin–mineral premix supplied the following per kilogram of diet: vitamin A, 1500 IU; vitamin D_3_, 200 IU; vitamin E, 10 mg; vitamin K3, 0.5 mg; thiamine, 1.8 mg; riboflavin, 3.6 mg; D-pantothenic acid, 10 mg; folic acid, 0.55 mg; pyridoxine, 3.5 mg; niacin, 35 mg; cobalamin, 0.01 mg; biotin, 0.15 mg; Fe, 80 mg; Cu, 8 mg; Mn, 60 mg; Zn, 40 mg; I, 0.35 mg; and Se, 0.15 mg

^b^ME = metabolizable energy

Starter-phase broiler chickens were housed in lighted coops, water was provided at all times, and continuous lighting was maintained. The temperature was maintained at 32 °C for the initial 5 d and then gradually reduced according to normal management practices, until a temperature of 22 °C was achieved. Broilers were reared on the ground under natural lighting during the finisher phase. Chickens were weighed at 1, 21 and 49 d of age to determine average daily gain (ADG). Daily feed consumption per replicate was recorded, the uneaten feed was discarded, and fresh feed was replenished. Average daily feed intake (ADFI) and the feed conversion ratio were then determined. At the end of the experiment, birds were randomly selected, deprived of feed for 12 h, weighed and killed. The abdominal fat pad, liver, pectoral and leg muscles were subsequently removed and weighed.

### Measurement of serum lipid parameters

Blood samples were allowed to clot at 4 °C and centrifuged at 1,500 × g for 10min before collecting the serum. Serum samples were stored at −20 °C. Concentrations of serum cholesterol (TC), triglyceride (TG), high-density lipoprotein cholesterol (HDL-C), low-density lipoprotein cholesterol (LDL-C) and non-esterified fatty acid (NEFA) content were measured by colorimetric enzymatic methods using commercial kits purchased from Nanjing Jiancheng Bioengineering Institute (Nanjing, China). Triiodothyronine (T_3_), thyroxine (T_4_), insulin (INS), free triiodothyronine (FT_3_), free thyroxine (FT_4_) and glucagon (GLU) were measured with the RIA kits provided by Beijing North Institute of Biotechnology (Beijing, China).

### Determination of hepatic lipid parameters assay

Total liver lipid content was determined on homogenized liver samples using a mixture of chloroform and methanol (2:1 v/v) according to the method of Folch [74]. The levels of hepatic TG and total TC content were determined using commercial kits (GPO-PAP and CHOD-PAP) purchased from the Nanjing Jiancheng Bioengineering Institute. The TG level in homogenates of liver was evaluated following the manufacturer’s protocols. Hepatic lipase activity and NEFA content was determined by using commercial kits from Nanjing Jianchen Biotechnology Institution. Approximately 100 mg of liver was homogenized on ice with 1 mL of physiological saline. An aliquot of these homogenates was used for protein determination by using commercial kits from Nanjing Biyuntian Biotechnology Institution and the remainder was centrifuged at 1,000 × g for 10 min at 4 °C. Clear supernatants were used for the assay. HL activity and NEFA concentrations were directly proportional to the colour intensity and were determined by measurement of absorbance at 550 and 440 nm, respectively. One unit of HL enzyme activity represents 1 mg tissue protein releasing 1 μmol of fatty acid per hour. Hepatic glycogen content was determined by using the commercial kit from Nanjing Jiancheng Biotechnology Institution.

### Real-time quantitative RT-PCR

Total RNA was extracted from liver using TRIZAOL reagent (Shengxing, China), according to the manufacturer’s prtocol and quantified by measuring the optical density in a photometer (Eppendorf Biophotometer) at 260 nm. The absorption ratios (260/280 nm) for all preparations were between 1.8 and 2.0. RNA sample aliquots were subjected to electrophoresis through a 1.4 % agarose formaldehyde gel to verify their integrity. The RNA concentrations were adjusted to 1 μg/μL by measuring the optical density (OD) value, and the samples were then stored at −80 °C.

Reverse transcription was performed using the method of Zhao S [75]. An aliquot of complementary DNA sample was mixed with 20μl SYBR_Green PCR Master Mix (Roche, Switzerland) in the presence of 10 pmol of each forward and reverse primers for ACC, FAS, PPAR-α, CPT-I, SREBP-1c, ACLY, AMPKα1,AMPKβ1, AMPKβ2,AMPKγ1, and then it was subjected to PCR under standard conditions (40 cycles). As an internal control, the same RT products were also subjected to chicken β-actin RNA. All samples were analyzed in duplicate using the IQ5 Sequence Detection System and programmed to conduct one cycle (95 °C for 1min) and 40 cycles (95 °C for 20 s, 60 °C for 30 s and 72 °C for 30 s). Result (fold change) was calculated using the 2-ΔΔCt method. 2-ΔΔCt = (Ct ij – Ct β-actin j) – (Ct i1 – Ct β-actin1), where Ct ij and Ct β-actin j are the Ct for gene i and for β-actin in a pool or a sample (named j) and where Ct i1 and Ct β-actin1 are the Ct in pool 1 or sample 1, expressed as the standard. The primers were designed by premier 5.0 software (Premier Biosoft International, Palo Alto, CA, USA) and the positions are referred to chicken for SREBP-1c, ACC, PPARα, CPT-I, AMPK, FAS, ACLY and β-actins (Table 7). The primers synthesized by Invitrogen Biological Company (Shanghai, China) were designed to flank known or putative introns, preventing amplification of any contaminating genomic DNA.

**Table 7:** Prime sequence of targeted genes and β-actin

Gene	GenBank acession number	Primer sequences (5′–3′)	Orientation	Product size (bp)
β-actin	L08165	TGCGTGACATCAAGGAGAAG TGCCAGGGTACATTGTGGTA	Forward Reverse	300
ACC	J03541	GTTGTGGTTGGCAGAGCAAG GCACCAAACTTGAGCACCTG	Forward Reverse	284
CPT-I	AY675193	GGGTTGCCCTTATCGTCACA TACAACATGGGCTTCCGTCC	Forward Reverse	151
FAS	NM205155	TGAAGGACCTTATCGCATTGC GCATGGGAAGCATTTTGTTGT	Forward Reverse	96
SREBP-1c	AY029224	GTCGGCGATCCTGAGGAA CTCTTCTGCACGGCCATCTT	Forward Reverse	105
ACLY	AJ851548	CACCCAGAGGTGGATGTTCT GTTGCAGGCCCAATGTTAGT	Forward Reverse	188
PPARα	AF470455	CAAACCAACCATCCTGACGAT GGAGGTCAGCCATTTTTTGGA	Forward reverse	64
AMPKα1	DQ302133	CAAGTAGTGTCTCGCACGGT GACTGATAGCTGGTCCCACG	Forward Reverse	133
AMPKβ1	DQ398947	CACCAAGGATGGGGACAGAC TTCCAGATCCTGCTGCCAAG	Forward Reverse	127
AMPKβ2	DQ640761	ACACTGCCTTCTTCTCCCTC GTGGGAGCTGAAAACACTGG	Forward Reverse	195
AMPKγ1	DQ133598	AGGACTCCTTCAAGCCGTTG AACTCAGGCTTTGGGACCTC	Forward Reverse	196

### Statistics analysis

The experimental data were expressed as the mean ± SE, and differences were considered significant when *P* < 0.05, as test by one-way analysis of variance (ANOVA) followed by a multiple range test, using the Statistical Packages for Social Science 16.0 and Excel 2003 in Microsoft. Replicate was considered as the experimental unit for the entire index determined.

## Abbreviations

(−)-HCA: (−)-hydroxycitric acid
ACC: acetyl CoA carboxylase
ACLY: ATP-citrate lyase
ADFI: average daily feed intake
ADG: average daily gain
AMPK: 5′-monophosphate-activated protein kinase
BW: body weight
CPT- I: carnitine palmitoyl transferase- I
FAS: fatty acid synthase
HDL-C: density lipoprotein-cholesterol
HL: hepatic lipase
LDL-C: low-density lipoprotein cholesterol
NEFA: non-esterified fatty acid
PPARα: peroxisome proliferators-activated receptor α
SREBP-1c: sterol regulatory element binding protein-1c
T_3_: triiodothyronine
T_4_: thyroxin
TC: cholesterol
TG: triglyceride
